# Artificial cornea transplantation and visual rehabilitation: an integrative review

**DOI:** 10.5935/0004-2749.2021-0350

**Published:** 2022-09-06

**Authors:** Tayná Meneses Fernandes, Mariana Cassiano Alves, Caroline Oliveira Diniz, Guilherme Cunha Queiroz, Sabrina T. Reis

**Affiliations:** 1 Curso de Medicina, Faculdade Atenas, Passos, MG, Brazil; 2 Universidade Federal dos Vales do Jequitinhonha e Mucuri, Teóflo Otoni, MG, Brazil; 3 Faculdade de Medicina, Universidade de São Paulo, São Paulo, SP, Brazil

**Keywords:** Corneal transplantation, Visual prosthesis, Cornea, Rehabilitation, Visual acuity, Transplante de córnea, Próteses visuais, Córnea, Reabilitação, Acuidade visual

## Abstract

Due to the development of complications and the biocompatibility and scarcity of transplant donor tissues, artificial corneas, which can be used for the rehabilitation of optical functions, have been developed. The current study aimed to analyze the visual rehabilitation effects of the Boston type I keratoprosthesis, Boston type II keratoprosthesis, Aurolab keratoprosthesis, osteo-odonto-keratoprosthesis, and tibial bone keratoprosthesis. Results showed that the Boston type I keratoprosthesis was the most effective for visual rehabilitation in patients with moist ocular surfaces. The Aurolab keratoprosthesis had a lower efficacy for visual rehabilitation. Nevertheless, it is still a viable option for individuals in economically restricted countries. In patients with dry eyes, the Boston type II keratoprosthesis was associated with the best visual rehabilitation. However, the final visual acuity of patients who received osteo-odonto-keratoprosthesis and tibial bone keratoprosthesis implantation was not evaluated as the necessary information was not available.

## Abstract (pt)

Em decorrência de complicações, da biocompatibilidade e da escassez de tecido doador para transplantes de córnea natural, foram elaboradas córneas artificiais que são potenciais para reabilitar funções ópticas. Nessa perspectiva, objetivou-se a análise da eficácia da reabilitação visual entre os implantes: Boston tipo I, Boston tipo II, Aurolab, osteo-odonto-ceratoprótese e ceratoprótese de Osso Tibial. De modo geral, a princípio observou-se uma tendência de melhoria da *Best-corrected visual acuity* em todos os tipos de lentes, mas considerável queda durante acompanhamento a longo prazo. O dispositivo com melhor reabilitação visual em pacientes com superfícies oculares úmidas é a Boston tipo I, seguida pela Aurolab, que é economicamente viável em países emergentes. Ao considerar pacientes com olhos secos, o implante de Boston tipo II apresenta maior reabilitação visual. Por fim, em virtude de não apresentarem dados equiparáveis, as lentes osteo-odonto-ceratoprótese e de osso tibial não puderam ser analisadas.

## INTRODUCTION

Approximately 441 million people worldwide have visual impairment, and 36 million are blind^(1)^. Vision-related issues can reduce a person’s quality of life^(2)^. Moreover, the risk of mortality increases by more than double due to the high incidence of accidents and the increasing number of falling events^(3)^. These phenomena can affect the economy owing to a decreased number of active workforce members, which is mainly associated with a lack of treatment access.

Corneal disease-related blindness is a factor influencing optical health^(4)^. The cornea is a squamous stratified epithelial tissue. Moreover, it comprises a convex transparent layer located on the anterior eye surface that protects the inner tissues and transmits light, thereby increasing the eye’s refractive capacity^(5)^. Complications in this structure can cause several degenerative, dystrophic, infectious, and inflammatory disorders affecting the ocular surface. In such cases, transplantation remains the primary method of visual rehabilitation. However, the availability of donor tissue is the main limiting factor in performing this procedure in emerging countries^(6)^.

Eye banks were established due to quality control demands for donated visual elements. These establishments are responsible for the removal, transport, evaluation, classification, preservation, storage, and availability of tissues^(7)^, including those used in corneal transplantation. Ophthalmologists are the end-users of these tissues as they are the ones who choose and use them based on their patients’ diagnoses^(8)^. Due to the lack of human tissue donors, artificial lenses have been used as alternative options for treating corneal diseases as they can improve visual acuity (VA) without exclusive dependence on donors.

Traditional corneal transplantation is the most commonly accepted treatment for vision restoration in patients with acute blindness^(9)^. Approximately 12.7 million people are on the waiting list for a procedure that requires ocular tissues, and only 1 in 70 cases is covered worldwide^(10)^. Currently, donation is the primary method by which transplant surgical materials are obtained. Thus, viable alternatives are important to meet the current procedural demands. To address the development of complications and the biocompatibility and scarcity of donor tissues, novel artificial corneas with transparent, non-toxic, and biomechanical properties have been established. They have optical functions and, thus, can be used in patients who are waiting for medical interventions^(11)^.

Therefore, considering the current increase in the prevalence of artificial corneal transplants, this study aimed to perform an integrative literature review to evaluate the efficacy and visual rehabilitation effects of the Boston type I keratoprosthesis (BKPro I), Boston type II keratoprosthesis (BKPro II), Aurolab keratoprosthesis (Auro KPro), osteo-odonto-keratoprosthesis (OOKP), and tibial bone keratoprosthesis (tibial bone KPro).

## METHODS

This article is an integrative review as it includes studies that used different methodologies. This type of review allows researchers to define concepts, review theories and evidence, analyze methodological problems, and synthesize a specific topic^(12)^. The current integrative literature review was started by identifying the topic of interest, followed by a database search of articles (via the use of descriptors and inclusion and exclusion criteria). Finally, the selected articles were investigated, and the information obtained was analyzed.

The following question was used to guide the study: What is the impact of artificial corneal transplantation on the rehabilitation of patients? Relevant studies were searched in PubMed and Biblioteca Virtual em Saúde. The *‘Descritores em Ciências da Saúde*’ (DeCS) was also used to define the descriptors. The search for articles was conducted in February 2021.

For the database search, the following descriptors were used: “Corneal grafting AND artificial cornea,” “Artificial cornea AND visual rehabilitation,” and “Artificial cornea AND postoperative period.” The following studies, which met the following inclusion criteria, were selected: 1) observational studies, controlled clinical trials, and randomized trials; 2) studies published within the last 5 years; and 3) articles that answered the guiding question. Meanwhile, the following studies were excluded: 1) integrative and systematic reviews, meta-analyses, and case reports and 2) studies that addressed the study question only in children or older people.

Four authors initially selected the articles for this review in an individual and standardized manner. Moreover, they aimed to select studies that followed the guiding question and met the pre-established inclusion criteria. In total, 126 published articles were found in the databases using the described descriptors and filters. All studies were verified and analyzed based on their titles and abstracts. However, those that did not answer the guiding question and those that have duplicates were excluded. Finally, 38 articles in MEDLINE were included.

In the second stage of selection, we performed a complete reading of the 38 articles. Subsequently, the researchers had a meeting and discussion, and only 25 studies were selected. Studies that only presented the corneal transplantation techniques and those that did not answer the guiding question after the whole reading were excluded.

**Figure 1:**
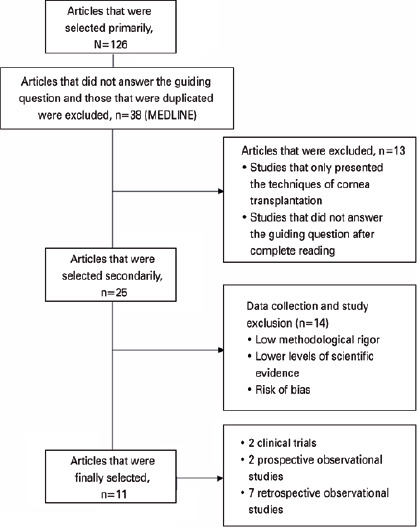
Articles selection process.

For the final selection, the instrument validated by Ursi (2005)^(12)^ for data collection was used. In total, 11 articles met the eligibility criteria, and they had the best methodological rigor and levels of scientific evidence and a low risk of bias. Among them, two were clinical trials (one controlled and another randomized controlled); two, prospective observational articles; and seven, retrospective observational studies (Figure 1).

Finally, for the critical analysis of 11 eligible studies, the Agency for Healthcare Research and Quality^(13)^ classification of scientific evidence levels was used. It covered six types of evidence, which were as follows: (I) evidence from meta-analyses and systematic reviews, (II) evidence from randomized clinical trials, (III) evidence from clinical trials without randomization, (IV) evidence obtained from cohort and case-control studies, (V) evidence from a systematic review of descriptive and qualitative articles, and (VI) evidence derived from descriptive or qualitative studies.

## RESULTS

BKPro I^(14,15,16,17,19)^ was used in six studies, the BKPro II^(20)^ in one, the Auro KPro and BKPro I in one^(21)^,the Auro KPro in one^(22)^, the OOKP in one, the tibial bone KPro in one^(23)^, and the BKPro I and BKPro II^(24)^ in one. Of these studies, five were performed in the USA^(14,15,17,19,20)^, two in India^(21,22)^, one in Canada^(16)^, one in the United Kingdom^(18)^, one in Spain^(23)^, and one in Ireland^(24)^. All articles were written in English.

Six articles were published in 2016^(14,16,17,20,22,24)^, one in 2017^(18)^, three in 2018^(15,19,22)^, and one in 2019^(21)^. All articles analyzed the use of artificial corneal keratoplasty.

Table 1 depicts the information collected. In addition, assessment was performed using data on retention, complications, and VA (Table 2).

**Table 1:** Data collected from other studies

Authors	Year/country	Study types	Evidence level/AHRQ	Lens types
Goins et al.^(14)^	2016/the USA	Retrospective observational study	2B/IV	Boston type I KPro
Aravena et al.^(15)^	2018/ the USA	Controlled clinical trial	1B/III	Boston type I KPro
Muzychuk et al.^(16)^	2016/CA	Controlled and randomized clinical trial	1B/II	Boston type I KPro
Rudnisky et al.^(17)^	2016/ the USA	Prospective observational study	2B/IV	Boston type I KPro
Ang et al.^(18)^	2017/ the UK	Prospective observational study	2B/IV	Boston type I KPro
Driver et al.^(19)^	2018/ the USA	Retrospective observational study	2B/IV	Boston type I KPro
Lee et al.^(20)^	2016/ the USA	Retrospective observational study	2B/IV	Boston type II KPro
Basu et al.^(21)^	2019/IN	Retrospective observational study	2B/IV	Boston type I KPro and Aurolab KPro
Venugopal et al.^(22)^	2016/IN	Retrospective observational study	2B/IV	Aurolab KPro
Charoenrook et al.^(23)^	2018/ES	Retrospective observational study	2B/IV	Osteo-odonto- keratoprothesis and Tibial bone keratoprothesis
Duignan et al.^(24)^	2016/IR	Retrospective observational study	2B/IV	Boston type I and II KPro

AHRQ= Agency for Healthcare Research and Quality.

**Table 2:** Evaluation of the procedures

Authors	Retention rate (%)	Complication rate (%)	Visual acuity BCVA of ≥20/200
Goins et al.^(14)^	85.3 (64/75)	Retroprosthetic membrane Maculopathy - 34.7	Final: 57.3 (43/75)
Aravena et al.^(15)^	74.3 (55/74)	Retroprosthetic membrane - 51.7	Mean: 86 (50/58) Final: 22 (18/50)
Muzychuk et al.^(16)^	24 months: (37/37 60 months: 96 (25/26)	Glaucoma - 65 Retroprosthetic membrane - 47	2 years: 57 (21/37) Final: 46 (12/26)
Rudnisky et al.^(17)^	93 (279/300)	NI	Mean: 84.7 (254/300) Final: 80.9 (241/300)
Ang et al.^(18)^	90 (59/66)	NI	3,5 years: 100 (66/66) 5-year estimative: 60 (40/66)
Driver et al.^(19)^	90 (207/231)	Retroprosthetic membrane - 40 e 51 Persistent epithelial defect - 37 e 24	1 year: 69.05 (145/210) Final: 41.07 (23/56)
Lee et al.^(20)^	50 (24/48)	Retroprosthetic membrane - 60.4	Mean: 91.7 (44/48) Final: 37.5 (18/48)
Basu et al.^(21)^	Boston type I KPro: 70.5 (55/78) Aurolab KPro: 62.5 (35/56)	Glaucoma - 28.4	Boston type I, mean: 87.3 (68/78) Final: 26.92 (21/78) Aurolab KPro, mean: 90 (49/56) Final: 26.78 (15/56)
Venugopal et al.^(22)^	73.3 (11/15)	Retroprosthetic membrane - 46.7 Graft infection - 26.7	Final: 60 (9/15)
Charoenrook et al.^(23)^	OOKP: 67 (97/145) Tibial bone keratoprothesis: (61/113)	Retinal detachment - 15 e 16 Retroprosthetic membrane - 3 e 23	NI
Duignan et al.^(24)^	Boston type I KPro: 85 (29/34) Boston type II KPro: 67 (2/3)	Retroprosthetic membrane - 52.9 Glaucoma - 17.6	Final: 82.4 (28/34)

BCVA= best-corrected visual acuity; NI= not indicated; OOKP= osteo-odonto-keratoprosthesis.

VA was defined as the ability of the eye to identify spatial details or to perceive the shape and contour of objects. It is essential for assessing the progression of eye diseases and therapy success. The measurement of VA with the Snellen chart is a method applied to diagnose vision function. Each line in the chart has a corresponding fraction. The first number of fractions indicates the distance in meters from the chart, and the second number represents the distance that a normal eye can see. According to the Snellen chart, the best-corrected VA (BCVA) is the best possible vision that an eye can achieve using glasses or contact lenses. With a BCVA of ≥20/200, the tested eye can see at 6 m (or 20 feet) what a normal eye can see at 60 m (or 200 feet).

The articles analyzed (n=11) included 1256 eyes and 1303 procedures. Among them, 923 utilized the BKPro I; 51, the BKPro II; 71, the Auro KPro; 145, the OOKP; and 113, the tibial bone keratoprosthesis. The mean follow-up time ranged from 9.65 to 114 months. The corneas used were either fresh or frozen^(16)^. The mean age of the patients ranged from 43 to 71 years, and majority were men. Visual rehabilitation was evaluated by analyzing different variables (Table 2), and the results are shown in table 3. Although it was necessary to convert corrected-distance VA (CDVA) to BCVA, one article did not have any data about BCVA^(25)^. Next, data were combined according to lens type to facilitate comparison. The percentages reported refer to the number of patients who obtained the expected outcomes during the study. However, one article did not report the mean or final VA of the patients. Hence, studies that used osteo-odon to-keratoprosthesis and tibial bone keratoprosthesis were not analyzed^(24)^.

**Table 3:** General results categorized according to lens types

Lens types	Sample	Retention rate (%)	Final visual acuity BCVA of ≥20/200 (%)	Mean follow-up
Boston type I Kpro	923	84 (773/923)	62,18 (426/685)	39.37 months
Boston type II Kpro	51	51 (26/51)	39,21 (20/51)	56.1 months
Aurolab Kpro	71	65 (46/71)	33,80 (24/71)	36.75 months
Osteo-odonto-keratoprosthesis	145	67 (97/145)	-	114 months
Tibial bone keratoprosthesis	113	54 (61/113)	-	50.4 months

Results showed a trend toward improvement in the initial BCVA in all lens types. However, with consideration of the follow-up durations, there were differences in terms of short- and long-term BCVA. Therefore, there was a significant trend between the follow-up durations and the final outcomes. That is, if the follow-up time was longer, the risk of device extrusion was higher, and the final VA was lower. Thus, the BKPro I was associated with the best visual rehabilitation. Moreover, patients who received BKPro I implantation had the highest retention rate (84%) and the best final VA (62.18%), and their follow-up time was only 39.37 months. The VA and retention rate of patients who received BKPro II implantation were 39.21% and 51%, respectively. Moreover, these patients had a long mean follow-up time (56.1 months). Finally, patients who received Aurolab KPro implantation had a low final VA (33.80%) and retention rate (65%), and a short average follow-up time (36.75 months). Based on the long-term analysis, the VA significantly decreased in all types of lenses.

## DISCUSSION

In this integrative review, we performed an analysis of visual rehabilitation using five types of artificial corneas, which were as follows: Boston type I, Boston type II, AuroLab, osteo-odonto-keratoprosthesis, and tibial bone keratoprosthesis. Boston type I lens had the best outcomes. That is, 62% of patients had a final VA of 20/200 and a retention rate of 84%. Its main complication was neuroprosthetic membrane (RPM). Similarly, Kanu et al. showed better rehabilitation outcomes as evidenced by VA improvement in 75% (51/68) of the studied eyes within 5 years and 66.7% (46/68) within 10 years, with a retention rate of 89.2%. The most common complication was RPM^(26)^. Another study revealed that 70% of the studied eyes achieved a VA of 20/200 or better within 3 months. However, after 60 months, only 44% maintained this acuity due to postoperative complications, particularly RPM^(27)^.

Keratoprosthesis implants, which were developed in 1968, are most commonly used worldwide, with more than 12,000 transplants performed to date. Over the years, according to the indications and related complications, this device type has undergone modifications to improve its outcomes. The BKPro I implant is recommended for individuals with corneal blindness whose eyes are still wet and can blink^(28)^. According to Homayounfar et al., who analyzed the use of BKPro I in elderly patients, a postoperative VA of 20/200 or better was achieved in 82% (36/44) of patients. Further, the final VA remained at 45.5% (20/44), and the device retention rate was 88.9%. Patients aged over 75 years old had excellent outcomes, and these are associated with a better quality of life and fewer long-term effects^(29)^. However, Fung et al. showed that implants were not recommended for children as they have a shorter distance between the lens and cornea, thereby making them more susceptible to serious complications and extrusion of implants such as BKPro I^(30)^. This procedure affects the visual rehabilitation and wellness of patients, particularly the longevity of these individuals.

Boston type II keratoprosthesis is used in patients with severe dry ocular surface disease, particularly in cases of Stevens-Johnson syndrome/toxic epidermal necrolysis (SJS/TEN). This device consists of modifying the BKPro I implant with an anterior bulge projected through eyelids that are surgically closed. However, only approximately 200 transplants have been performed until December 2015^(20,31)^. According to our results, visual rehabilitation was achieved in 39.21% (20/51) of patients who used this lens, with a final VA of 20/200 or better. The retention rate was 51% (26/51), and the mean follow-up time was 56.1 months. Further, the most common complication was RPM. However, according to Iyer et al., the final VA was achieved in 70% (11/16) of patients after Boston type II keratoprosthesis implantation at a median follow-up of 33 months. The retention rate was 90%, and RPM was not the most common recurrent complication^(32)^. The discrepancy between the results can be explained by the relatively short follow-up time and the small sample size. According to Lee et al., this artificial cornea had limited outcomes^(20)^.

Owing to limited resources and reduced accessibility to BKPro I implants, the AuroLab keratoprosthesis was developed in India in 2011. The design of this lens is similar to that of BKPro I, which comprises a faceplate, locking ring, and backplate made of polymethylme-thacrylate. The AuroLab implant is a low-cost device, costing only $100. Some studies have indicated that the outcomes of Auro KPro are comparable to those of BKPro I. However, it still has some deficiencies ^(21,33)^. In our review, 33.8% (24/71) of patients who had this lens had a final VA of 20/200 or better, and the retention rate was 65% (46/71) during a mean follow-up of 36.75 months. The main complications were RPM and glaucoma. However, Sharma et al. showed that 60% (6/10) of patients had a VA of 20/200 or better for 1 year, and the retention rate was 90% (9/10). This study showed VA worsening over time, mainly due to complications such as inflammatory dendrites. In addition, these authors confirm the need to conduct a long-term follow-up study on a large sample^(33)^, which may justify the discrepancy in our results.

The OOKP is a device built from the patient’s tooth, and it acts both as a biological tissue and artificial structure^(23)^. It was developed in 1963 by Strampelli^(34)^ and later modified by Falcinell^(35,36)^. The procedure is complex, and it occurs in two stages, with a long operative period. Follow-up is a lifelong process in patients with this device, and patients must be committed to continuous postoperative care and periodic consultations. OOKP is indicated for eyes with severe dryness, with no eyelids or blinking due to damaged ocular surfaces. Thus, it can help in the recovery of a sustainable VA^(37)^. The current review did not assess the final VA of this lens because the necessary information was not available in the study. However, it had a median maximum VA of 20/250 in 18% of cases and a follow-up of over 114 months^(23)^. In a study of OOKP in patients with severe chemical and thermal burns, the authors showed that the final VA of 43% (6/14) of patients with 5 years was 20/200, and the retention rate was 85% (11/14)^(38)^. By contrast, in our study, the retention rate was only 67% (97/145). The most common complications were RPM, glaucoma, and retinal detachment. However, according to Afonso et al., glaucoma was the most prevalent.

In some patients, particularly elderly ones, the existing teeth with which to perform OOKP implantation are inadequate or nonexistent^(39)^. Based on this perspective, in 1985, Trempano implemented a small tibial bone disc implant, referred to as the tibial bone keratoprosthesis^(40)^. Similar to OOKP, this device remains in the inferior infraorbital region for 3 months to facilitate vascularization and biocompatibility. Moreover, it is implanted in patients during the second surgical stage. The tibial bone KPro is indicated for patients with opacification of the cornea and severe ocular surfaces, in whom keratoplasties could not be successful^(23)^. The aforementioned study did not report the final VA of the patients, with only a median maximum VA of 20/50 in 23% of patients within 50.4 months. However, Charoenrook et al. recorded a VA of 20/400 within 5 years (33%) and a retention rate of 69.5%^(39)^. In our study, the retention rate was 54%, and the main complications were RPM, glaucoma, and retinal detachment.

The current study had some limitations. It only included few studies on specific devices, owing to the recent emergence of these procedures. The studies had heterogeneous follow-up times, thereby making a reliable comparison challenging. In addition, the studies included small samples that restricted a comprehensive assessment due to the high costs of the procedures and low implementation rates. Notably, most studies analyzed were observational in nature. Therefore, there is a need to perform experimental studies such as randomized clinical trials and those with larger sample sizes.

In conclusion, the BKPro I device had the most significant potential for visual rehabilitation in cases of moist ocular surfaces. However, although the Auro KPro had low visual rehabilitation outcomes, it is a viable option in economically restricted countries. In contrast, when considering dry eyes, the Boston type II lens had the best visual rehabilitation. Moreover, several patients who used keratoprostheses achieved visual capacity recovery. However, these devices require long-term improvements as the rehabilitation rates are still disproportionate to time.
